# Selinexor With Anti-PD-1 Antibody as a Potentially Effective Regimen for Patients With Natural Killer/T-Cell Lymphoma Failing Prior L-Asparaginase and PD-1 Blockade

**DOI:** 10.1093/oncolo/oyad241

**Published:** 2023-08-24

**Authors:** Rong Tao, Chuanxu Liu, Wenhao Zhang, Yang Zhu, Yujie Ma, Siguo Hao

**Affiliations:** Department of Hematology, Xinhua Hospital, Shanghai Jiao Tong University School of Medicine, Shanghai, People’s Republic of China; Department of Lymphoma, Fudan University Shanghai Cancer Center, Shanghai, People’s Republic of China; Department of Hematology, Xinhua Hospital, Shanghai Jiao Tong University School of Medicine, Shanghai, People’s Republic of China; Department of Lymphoma, Fudan University Shanghai Cancer Center, Shanghai, People’s Republic of China; Department of Hematology, Xinhua Hospital, Shanghai Jiao Tong University School of Medicine, Shanghai, People’s Republic of China; Department of Lymphoma, Fudan University Shanghai Cancer Center, Shanghai, People’s Republic of China; Department of Hematology, Xinhua Hospital, Shanghai Jiao Tong University School of Medicine, Shanghai, People’s Republic of China; Department of Hematology, Xinhua Hospital, Shanghai Jiao Tong University School of Medicine, Shanghai, People’s Republic of China; Department of Hematology, Xinhua Hospital, Shanghai Jiao Tong University School of Medicine, Shanghai, People’s Republic of China

**Keywords:** natural killer/T-cell lymphoma, selinexor, PD-1 blockade, central nervous system involvement

## Abstract

**Background:**

Natural killer/T-cell lymphoma (NKTCL) is a rare and heterogeneous tumor type of non-Hodgkin’s lymphoma (NHL) with a poor clinical outcome. There is no standardized salvage treatment failing l-asparaginase-based regimens. Here we report our retrospective results of the combined use of selinexor and PD-1 blockade (tislelizumab) in 5 patients with NKTCL who had exhausted almost all available treatments.

**Patients and methods:**

A total of 5 patients with relapsed/refractory(R/R) NK/T-cell lymphomas failing prior l-asparaginase and anti-PD-1 antibody were retrospectively collected. They were treated with at least one cycle of XPO1 inhibitor plus the same anti-PD-1 antibody. Anti-PD-1 antibody (Tislelizumab) was administrated at 200 mg on day 1 every 3 weeks and selinexor doses and schedules ranged from 40 mg weekly for 2 weeks per 21-day cycle to 60 mg weekly per cycle.

**Results:**

Five patients with relapsed NKTCL with extensive organ involvement including 4 central nervous system (CNS) infiltration patients were included. Four patients achieved objective responses including 3 complete responses (CR) and 1 partial response (PR). After a median follow-up time of 14.5 (range, 5-22) months, 1 patient was still in remission with CR, and the other 4 patients discontinued due to disease progression with a median progression-free survival (PFS) of 6 months and median overall survival (OS) of 12 months. Four patients with CNS involvement achieved a median OS of 8 months. Our data suggest that selinexor in combination with an anti-PD-1 antibody is a promising small molecule and immunotherapy combination regimen for patients with relapsed or refractory NKTCL.

## Teaser

This article reports retrospective results of the combined use of selinexor and PD-1 blockade (tislelizumab) in 5 patients with natural killer/T-cell lymphoma who had exhausted almost all available treatments.

**Implications for Practice**

The results of this study suggest that the combination of selinexor, and anti-PD1 antibody is a potentially effective treatment for patients with advanced, refractory NKTCL, even in cases of central nervous system involvement and prior resistance to PD-1 blockade. Clinicians should consider this treatment option for patients with NKTCL who have failed prior checkpoint inhibitors. Additionally, the study’s findings may have broader implications for the use of XPO1 inhibitors in the immunotherapy of other types of cancer, which should be further explored in future research.

## Introduction

Natural killer/T-cell lymphoma (NKTCL) is a rare and aggressive extranodal malignancy invariably with Epstein–Barr virus (EBV) infection. It is more frequently seen in East Asia and Latin America population. Currently, NKTCL is typically treated with l-asparaginase-based regimens with or without radiotherapy in a frontline setting. However, more than 40% of patients experience treatment failure.^1^ Relapsed/refractory patients had poor survival with a median progress-free survival (PFS) of 4.1 months and overall survival (OS) of 6.4 months.^2^ Immune checkpoint inhibitors (ICIs) have shown promising efficacy in NKTCL as NKTCL has a higher expression of PD-L1 induced by EBV infection and escapes the immune surveillance.^3^ However, upon their failure, there is no effective salvage treatment, and the prognosis is extremely poor.

The exportin 1 (XPO1) selective inhibitor selinexor has been approved by the US FDA for the treatment of relapsed or refractory diffuse large B-cell lymphoma. Early clinical trials included many subtypes of non-Hodgkin’s lymphoma including several T-cell lymphomas suggested some promising signals.^4^ Prior studies demonstrated that selinexor blocked the nuclear export of several transcription factors and increased the expression of the PD-1 in normal leukocytes and PD-L1 in tumor cell lines, which may sensitize the activities of ICIs. The combination therapy of selinexor and PD-1/PD-L1 blockade exerted superior anti-tumor activity than either therapy alone in animal models.^5^ Moreover, preclinical studies demonstrated that EBV SM protein’s nuclear exportin is mediated by XPO1 and XPO1 inhibitor could suppress the replication of EBV, a favorable signal for the control of EBV-driven NKTCL.^6,7^

Based on this clinical and biological rationale, selinexor in combination with PD-1 blockade may be a potent salvage regimen in NKTCL. Herein we report our experience with the combination use of selinexor and PD-1 blockade (tislelizumab), in NKTCL refractory to l-asparaginase and the same PD-1 blockade.

## Methods

### Patients and Treatment

A total of 5 patients with relapsed/refractory(R/R) NK/T-cell lymphomas failing prior L-asparaginase and anti-PD-1 antibody at Xinhua Hospital, Shanghai Jiao Tong University School of Medicine (Shanghai, China) since 2021, were retrospectively collected. They were treated with at least one cycle of XPO1 inhibitor plus the same anti-PD-1 antibody. Tislelizumab was administrated at 200 mg on day 1 every 3 weeks and selinexor doses and schedules ranged from 40 mg weekly for 2 weeks per 21-day cycle to 60 mg weekly per cycle (Table 1). The patients provided their written informed consent under a named patient program.

**Table 1.** Selinexor in combination with anti-PD1 antibody to treat 5 patients with refractory NKTCL.

Patient characteristics	Case 1	Case 2	Case 3	Case 4	Case 5
Age/sex	53/male	35/male	55/male	44/male	38/male
Staging at diagnosis	IV	II	I	II	I
Primary chemotherapy (cycles)	MEDA (6)	SMILE (4)	COEPL (6)	MESA (4)	GemLasp (4)
Primary radiotherapy (Gy)	No	Yes (54Gy)	Yes (50Gy)	Yes (50Gy)	Yes (50Gy)
Response to primary treatment	PR	CR	CR	CR	CR
Time from diagnosis to first progression (month)	11	14	7	22	11
Number of prior therapies	4	3	3	3	3
Refractory to pegaspargase	Yes	Yes	Yes	Yes	Yes
Refractory to anti-PD-1 antibody	Yes	Yes	Yes	Yes	Yes
Staging before trial treatment	IV	IV	IV	IV	IV
ECOG before trial treatment	4	3	2	3	2
Sites involved before trial treatment	Spinal cord, lumbar nerve roots, CSF	Sinus, orbit, frontal lobe, CSF	Testis, BM, cerebellum	Lymph nodes, extensive skin, muscles	Extensive skin and muscles, lymph nodes, bone, CSF
CSF cytology	Positive	Positive	Positive	N/A	Positive
CSF EBV-DNA	Positive	Positive	Positive	N/A	Positive
Plasma EBV-DNA before selinexor treatment (copies/mL)	1500	2400	5100	16 000 000	16 000
PD-L1 expresssion on tumor cells (IHC)	80%	40%	50%	30%	20%
Gene mutations detected by NGS	TP53, HLA-A, SPEN, JAK1	SPEN, ARID1A	JAK3, KMT2D, BCOR, KRAS	N/A	BCOR, TET2, IFNGR2
Time from diagnosis to selinexor treatment (month)	23	22	16	29	27
*Treatment*					
Cycle	21-day				
Anti-PD-1 antibody	200 mg, day 1				
Selinexor	40 mg day 1, 8 (C1 and C2) 60 mg day 1, 8 (C3 and thereafter)	40 mg day 1, 8	40 mg day 1, 8, 15	40 mg day 1, 8	60 mg day 1, 8, 15
*Outcomes*					
Adverse events	Fatigue (G1)	Fatigue (G1)	Fatigue (G1) vomiting (G1) hyponatremia(G1)	Neutropenia (G1) thrombocytopenia (G1)	neutropenia (G3) thrombocytopenia (G3) fatigue (G2) decreased appetite (G2) weight loss (G1)
Overall response	CR	CR	PD	CR	PR
CSF cytology	Negative	Negative	Positive	/	Negative
CSF EBV-DNA	Negative	Negative	Positive	/	Negative
Plasma EBV-DNA	Negative	Negative	Positive	Negative	Negative
Current outcome	Alive, NED	Dead from disease	Dead from disease	Alive with disease	Dead from disease
PFS time (month)	22.0	6.0	1.5	12.5	4.5
Continue in treatment	Yes	No	No	Yes	No
Survival time (month)	22.0+	12.0	5.0	14.5+	8.0

Abbreviations: MEDA: methotrexate, etoposide, dexamethasone, and pegaspargase. SMILE: dexamethasone, methotrexate, ifosfamide, l-asparaginase, and etoposide. COEPL: cyclophosphamide, vincristine, etoposide, prednisone, and pegaspargase. MESA: methotrexate, etoposide, dexamethasone, and pegaspargase. GemLasp: gemcitabine and pegaspargase. ECOG: eastern cooperative oncology group. CSF: Cerebrospinal Fluid. EBV: Epstein-Barr virus. NGS: next-generation sequencing. C: cycle. G: grade. CR: complete response. PD: progressive disease. PR: partial response. NED: no evidence of disease. PFS: progression-free survival.

### Response Assessment

18F-fluorodeoxyglucose (18FDG) positron emission tomography/computed tomography (PET/CT) was performed for systemic disease evaluation according to the International 2014 International Working Group criteria.^8^ Patients with central nervous system (CNS) involvement received additional contrast-enhanced magnetic resonance imaging (MRI), cerebrospinal fluid (CSF) examination and ophthalmologic examination if needed, for CNS disease evaluation per the International Primary CNS Lymphoma Collaborative Group response criteria.^9^ Response definition took into consideration both the International 2014 International Working Group criteria and the International Primary CNS Lymphoma Collaborative Group response criteria. As this was a retrospective study, schedule of PET/CT and MRI varied, but PET/CT evaluation after 4 cycles and MRI assessment every 1-2 cycles were performed in most cases. Plasma or CSF EBV-DNA copies were dynamically monitored by quantitative polymerase chain reaction (PCR). Adverse events were graded using the National Cancer Institute Common Terminology Criteria for Adverse Events, version 5.0.

## Results

### Patient Characteristics

The 5 patients were all male and the median age was 38 (range, 35-55) years. Before starting this designed treatment, they were diagnosed with stage IV NKTCL with extensive organ involvement, including 4 CNS infiltration patients. The median time from the initial diagnosis to the treatment was 23 (range, 16-29) months. 3 patients had an Eastern Cooperative Oncology Group (ECOG) performance status (PS) 3 or 4. 4 patients received 3 prior lines of therapy and one patient received 4 prior lines of therapy. l-asparaginase-based chemotherapy with or without radiotherapy was the front-line therapy. Four patients received l-asparaginase-based chemotherapy with radiotherapy, 3 of them treated with chemotherapy sandwiched with radiotherapy and one treated with sequential chemoradiotherapy. Disease progression after primary treatment was observed in no more than 10 months for all 5 patients and then the treatment failure of combination therapy with anti-PD-1 antibody-based ICIs was also noted in these patients in subsequent treatments. Other novel or CNS-directed drugs like chidamide, brentuximab vedotin, high dose-methotrexate, temozolomide, and pemetrexed also failed for all patients. The details of patient characteristics are summarized in Table 1.

### Response and Survival Outcome

**Figure 1.**
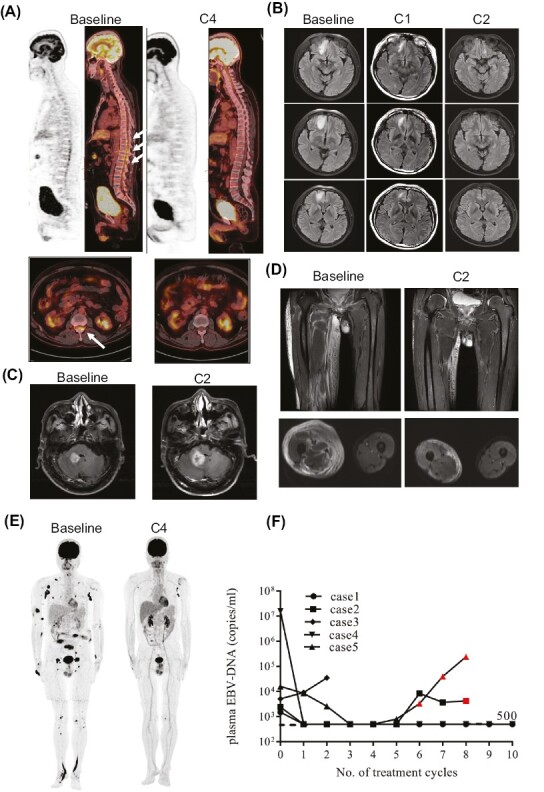
Radiological evaluation of response and dynamic monitoring of plasma EBV-DNA copies after treatment with selinexor and anti-PD-1 antibody. (**A**) PET/CT showed lumbar multiple nerve roots and spinal cord involvement in patient 1 (baseline), and complete remission was achieved after 4 cycles of treatment (C4). (**B**) MRI-enhanced T2 FLAIR showed that the patient’s right frontal lobe was involved (baseline), and the lesion was significantly reduced after one cycle of treatment (C1) and disappeared after 2 cycles of treatment (C2). (**C**) MRI showed new lesions in the cerebellum after 2 cycles of treatment. (**D**) MRI showed swelling in the right leg shrank significantly after 2 cycles of treatment. (**E**) PET-CT showed that the patient had extensive skin, soft tissue, lymph nodes, and bone involvement (baseline). After the fourth course of treatment, most of the lesions resolved, and only bilateral axillary lymph nodes were left with increased FDG metabolic uptake (C4). (**F**) The plasma EBV-DNA in case 1 decreased to undetectable (in our hospital less than 500 copies/mL is undetectable) and remained undetectable in the following treatment. The plasma EBV-DNA in case 2 decreased to less than 500 copies/mL after 1 cycle of the treatment but increased after 6 cycles. Case 3 patient with no response also did not see a decrease in EBV-DNA level. The EBV-DNA in case 4 decreased to undetectable status after 1 cycle and remained undetectable before disease progression. The EBV-DNA in case 5 can still achieve undetectable after 3 cycles but increased after radiological proved disease progression. * the red dots of cases 2 and 5 in the above graph: plasma EBV-DNA copies after disease progression.

**Figure 2.**
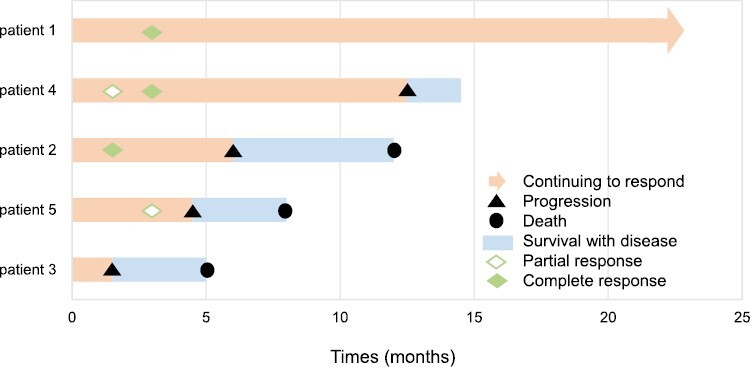
Swimmer plot illustrating a summary of clinical courses for the 5 patients after treatment with selinexor and anti-PD-1 antibody.

After a median follow-up time of 14.5 (range, 5-22) months, a median of 8 (range, 2-28) cycles of selinexor and tislelizumab were administered, and the objective response was observed in 4 patients including 3 complete responses (CR) and 1 partial response (PR) (Fig. 1A-1E). One patient was still receiving the combination treatment in CR and the other 4 patients discontinued due to disease progression (Fig. 2). Plasma EBV-DNA ranged from 1500 to 16 000 000 copies/mL before the combination treatment and all decreased to undetectable for 4 responders except for the one with progressive disease (Fig. 1F). The 3 patients with positive CSF EBV-DNA copies also experienced a negative conversion of EBV-DNA level in CSF samples. The plasma EBV DNA was correlated with response to the combination treatment and increased in 2 patients after disease progression.

### Adverse Events

All 5 patients experienced treatment-related AEs such as thrombocytopenia, neutropenia, and fatigue but the majority were grade 1 or 2. Only case 4 experienced grade 3 hematological toxicity neutropenia and thrombocytopenia and he also used the highest accumulative doses of selinexor per cycle compared to other patients. Immune-related toxicities were not observed. No patients discontinued the combination regimen because of treatment-related AEs and all the adverse events were manageable and resolved with supportive care (Table 1).

## Discussion

This is the first report of combined selinexor and PD-1 blockade for NKTCL. Five patients with the refractory disease to l-asparaginase-containing regimens and PD-1 blockade had exhausted almost all available treatment options but achieved rapid symptom remission and durable responses with a median PFS of 6 months and median OS of 12 months. Among them, 4 patients with CNS involvement achieved a median OS of 8 months. The first patient with relapsed spinal cord, lumbar nerve roots, and CSF involvement presented with limb numbness and lower-extremity weakness. Remarkably, after 1 week of this regimen, the patient’s limb numbness resolved and was able to stand up and walk and continued in CR for 22 + months. Case 2 presented with relapsed right frontal lobe and CSF involvement achieved CR after 2 cycles of selinexor and anti-PD-1 antibody and his neurological symptoms (eyes motility disorder and headache) resolved after just one cycle. The patient continued in response for 8 cycles of treatment but died 6 months after CNS progression. Case 3 showed progressive disease to 2 cycles of this regimen with cerebellum lesions enlargement and died 3 months later even with salvage whole-brain radiation therapy. Case 5 experienced extensive organ involvement including skin, muscles, lymph nodes, bones, and CSF during prior treatment. Reduction in skin nodules and improved lower-extremity weakness were observed after the first cycle of treatment. After 4 cycles, PET-CT evaluation showed a strong partial metabolic response (PMR). The patient continued treatment for 6 cycles before he progressed with peripheral recurrence and died 3.5 months later due to hemophagocytic lymphohistiocytosis. Patients with relapsed NKTCL and CNS involvement were reported to have a median OS of 3.8 months,^10^ and all 4 patients have substantially surpassed this time point. Seventy lymphoma-relevant genes using next-generation sequencing (NGS) were collected in 4 patients (except for case 4; Table 1). The only patient with no response to the combination regimen was detectable with JAK3, KMT2D, BCOR, and KRAS mutation. TP53 mutation was found in case 1 with CR. Due to limited cases, we have not observed any predispositions between mutation profiles and the response to the combination therapy.

The standard treatment for R/R NKTCL has not been established. Novel agents such as anti-CD38 antibody daratumumab,^11^ selective histone deacetylase inhibitor chidamide,^12^ aurora kinase inhibitor alisertib,^13^ and anti-CD30 antibody conjugate brentuximab vedotin^14^ were reported in a few cases with limited activities. ICIs may benefit patients with NKTCLdue to their disease properties. Anti-PD-1 antibody pembrolizumab for R/R NKTCL provided an ORR of 42.8%-100% in 3 retrospective studies^1^ but 2 prospective phase II trials using PD-1 blockade (sintilimab) and PD-L1 blockade (avelumab) in R/R NKTCL terminated early due to many patients had disease progression at the first evaluation.^15,16^ The ORR of sintilimab was 75.0% with a median duration of response (DOR) of 4.1 months and the ORR of avelumab was 38%. These results suggest that single-agent anti-PD-1/PD-L1 antibodies had modest activity in NKTCL and there is an urgent need to improve the efficacy of ICIs in NKTCL and reduce time to response. Overcoming drug resistance also remains significant challenges. Possible mechanisms of resistance to ICIs were associated with an inadequate generation or activation of anti-tumor T cell or memory T cell.^17^ Preclinical studies showed that selinexor could increase the expression of immune checkpoints in normal leukocytes and tumor cells. Concurrently administered selinexor and anti-PD-1 antibody led to superior tumor growth suppression to either therapy alone. Besides, when selinexor was combined with anti-PD-L1 antibodies, it increased the proportions of NK cells, CD4^+^ cells, and CD8^+^ CD62L^+^ CD44^+^ T cells (early activation and central memory-like phenotype).^5^ Our study showed promising results, with durable responses and rapid disease control observed in 4 patients, which support that combination therapy of selinexor and anti-PD-1 antibodies had a possible synergistic effect or even reversal of PD-1 blockade resistance in this disease. After experincing disease progression in the front-line therapy, case 1 received 2 cycles of temozolomide, lenalidomide, and tislelizumaband 4 cycles of tislelizumab and brentuximab vedotin in subsequent treatment. No response and a short-termresponse of improved symptom control but quick progression were observed, respectively, in this patient. He achieved CR after using selinexor plus tislelizumab and remained CR for at least 22 months. The best response of anti-PD-1 antibody-based combination therapy in case 2 was PR lasting for 4 months and he achieved CR with the combination of selinexor and PD-1 blockade for 6 months. Cases 3, 4, and 5 with acquired resistance to anti-PD-1 antibody showed no response, complete response, and partial response to the combination of selinexor and PD-1 blockade, respectively. The response to the combination regimen of selinexor and anti-PD-1 antibodies we observed was not correlated to the patient’s prior response to the same immune checkpoint inhibitors. As it was a retrospective study, we did not test the PD-L1 expression and changes in immune cell populations before redosing the same anti-PD-1 antibody and selinexor. It limited our further clarification of the possible mechanism for overcoming PD-1 blockade resistance. Another small sample size clinical trial using selinexor in combination with Gemox (gemcitabine and oxaliplatin) in 7 R/R patients with NKTCL achieved an ORR of 57% and a DOR of 3.1 months.^18^ In this preliminary study, an anti-PD-1 antibody with selinexor appears to be an attractive and effective small molecule and immunotherapy combination.

Anti-PD-1 antibody tislelizumab was administered at the recommended dose and schedule 200 mg day 1 per cycle in these 5 patients, while selinexor doses and schedules ranged from 40 mg weekly for 2 weeks per 21-day cycle to 60 mg weekly per 21-day cycle. A similar study using selinexor in combination with pembrolizumab in 25 patients with advanced melanoma adopted the selinexor starting dose of 60 mg twice weekly and pembrolizumab 240 mg every 3 weeks and 3 patients discontinued the regimen due to adverse events.^19^ Sixty milligrams were the recommended phase II dose of selinexor from phase I studies in non-Hodgkin’s lymphoma and solid tumors^4,20^, which was confirmed by the XPO1 target occupancy experiment.^21^ A further 3 + 3 design clinical trial found the maximum tolerated dose of selinexor was 40 mg on days 3, 5, and 7 when combined with chemotherapy (dexamethasone, ifosfamide, carboplatin, etoposide) in patients with R/R peripheral T-cell or NK/T-cell lymphoma.^22^ Overall, our study used a relatively lower dose of selinexor with a well-tolerated safety profile. Both cases 2 and 4 administered with selinexor of 40 mg weekly for 2 weeks every 3 weeks achieved CR and a higher rate of adverse events was observed in case 5 using selinexor of 60 mg weekly. But optimal doses and dose schedules need further clinical assessment.

## Conclusion

We observed impressive activity and manageable toxicity with the combination of selinexor and PD-1 blockade in patients with refractory NKTCL failing l-asparaginase and the same anti-PD-1 antibody. Further work is needed to elucidate the exact mechanism of action for combination therapy of selinexor and anti-PD-1 antibody and these data support a planned phase Ib/II study with the combination therapy in R/R NKTCL patients(NCT04425070).

## Data Availability

The data that support the findings of this study are available from the corresponding author upon request.
